# Combatting the occurrence of needle-stick injuries in a medical school: why is it still an issue?

**DOI:** 10.1186/s12909-024-05309-1

**Published:** 2024-03-20

**Authors:** Franca Keicher, Janina Zirkel, Tobias Leutritz, Sarah König

**Affiliations:** 1 Department of Paediatrics, University Hospital Würzburg, Würzburg, Bavaria Germany; 2 Department of Internal Medicine II, University Hospital Würzburg, Würzburg, Bavaria Germany; 3 University Hospital Würzburg, Institute of Medical Teaching and Medical Education Research, Würzburg, Bavaria Germany

**Keywords:** Needle-stick injuries, Medical students, Occupational health, Didactic intervention

## Abstract

**Background:**

Needle-stick injuries (NSIs) pose a safety risk for healthcare workers with great potential for serious infections. The aim was to determine numbers and causes of NSIs as well as the frequency with which medical students report NSIs in the final stages of study.

**Methods:**

An online questionnaire was developed and made available in January and February 2023 to all undergraduate medical students (*n* = 423) in the last 1.5 years of their degree course at Würzburg University, Germany.

**Results:**

The response rate was 19.6% (*n* = 84). Among respondents, 27.4% (*n* = 23) reported at least one NSI. Occurrence was particularly frequent in surgery, obstetrics and gynaecology, and internal medicine. Assisting with procedures, suturing, and blood sampling were considered high-risk activities. Lack of concentration, distraction, and time pressure played a role in incidents. Respondents did not report 18.8% of NSIs with the main reasons being fear of the consequences, self-assessment of the injury as minor, or the opinion of supervisors that reporting was unnecessary. Students with previous practice on simulators or patients were significantly more likely to suffer NSIs. Instructions from occupational health specialists beforehand correlated with fewer NSIs.

**Conclusion:**

We assume that trained students are more experienced in handling invasive procedures, leading to a greater adoption of corresponding activities and thus an increased risk of injuries in absolute numbers. This does not counter the need for didactic interventions prior to workplace-based training to raise awareness of NSI risks. Simultaneously, concepts must be developed and implemented to support reporting and alleviate fears regarding consequences.

## Background

Needle-stick injuries (NSIs), sometimes referred to as sharps injuries, pose a significant occupational risk to all healthcare workers, including medical personnel. The term NSI refers to “any puncture, cut or scratch injury to the skin caused by cannulas, scalpels, etc. that were contaminated with patient material, regardless of whether the wound bled or not - including direct contact with the skin or mucous membranes of the mouth, nose, and eyes” [1]. NSIs are known to occur frequently in clinical settings. For example, a cross-sectional study in Israel revealed that 53% of healthcare workers reported to have experienced at least one NSI in the last 5 years [2]. In Germany, the prevalence per year has been estimated to be 31.4%, with physicians being at greatest risk of experiencing NSIs [3]. Undergraduate medical students particularly during their work-place-based training, frequently engage in invasive procedures. As such, several studies have reported varying prevalence rates of NSIs among medical students, ranging from 21.4 to 59% [1, 4, 5].

NSIs can result in the transmission of various infections, including hepatitis B virus (HBV), hepatitis C virus (HCV), and human immunodeficiency virus (HIV). The risk of seroconversion for an unvaccinated person after NSI involving a confirmed hepatitis-B patient is highest, ranging from 23 to 62%. This is followed by the risk of transmission of HCV (0–7%) [6–8] and HIV (mean risk: 0.3%) depending on the form of exposure [9]. The timely reporting of NSIs is crucial, especially in cases of suspected HIV transmission, as it allows for appropriate post-exposure prophylaxis [10]. However, research indicates that a significant number of healthcare workers fail to report their NSI. Studies have reported a wide range of underreporting rates, varying from 30% to as high as 90% [11–14]. Studies of NSI reporting in medical students similarly revealed underreporting rates ranging from 29 to 53% [5, 14, 15]. This may be attributed to factors such as a lack of risk awareness, misperception of the seriousness of NSIs, and insufficient knowledge regarding reporting procedures [4, 15].

To prevent NSIs, a comprehensive approach involving strategies focused on educational interventions providing training and raising awareness has to be adapted. This includes notable emphasis on the use of materials with some safety mechanism, exemplified by the introduction of a law in Germany in 2013 mandating the use of safety instruments in procedures carrying a risk of infection resulting from NSIs [16]. Some publications report on successful curricular strategies in teaching. Seng et al. conducted a study in Singapore, in which simulation-based training, enhanced clinical experience, and improved knowledge regarding management and reporting procedures for medical students was implemented. Over a nine-year period, this approach resulted in a significant reduction in NSIs [17]. In another study, Calabro et al. aimed to evaluate the effects of a customized intervention on infection control. Based on a pre-test and focus group interviews, their design comprised a handwashing exercise, a lecture, and two case studies. Although this combination successfully increased participants’ knowledge, the study did not examine whether this approach resulted in a reduction in NSIs or higher rates of reporting [18]. A review study revealed low- to very low-quality evidence that education and training may cause small decreases in the incidence of sharps injuries two to 12 months after such an intervention [19]. New strategies, including the utilization of technologies such as virtual reality, have shown promise. These innovative approaches already demonstrated their effectiveness in promoting safety practices and raising awareness [20].

Despite the literature already available on NSIs, there is a lack of clarity regarding the frequency of these events in workplace-based training during the later stages of medical degree programmes. Consequently, the objective of this paper is to examine the incidence and underlying factors associated with NSIs among medical students, along with their reporting practices, with the ultimate goal of identifying potential educational strategies for prevention. By addressing these knowledge gaps, the outcomes of this study will contribute to improving the safety and overall well-being of medical students throughout their training.

## Methods

### Study design and participants

We conducted this prospective cross-sectional study in winter 2022/23 at a medical school in Germany offering a standard six-year curriculum comprising two pre-clinical years, three clinical years and one practical year (PY) of training. For this study, medical students were invited to participate while enrolled in various forms of workplace-based training: clinical rotations (CR) during the fifth year of medical school and during the PY. Of note, CR were mandatory in the specialties of surgery, internal medicine, gynaecology and obstetrics, paediatrics and adolescent medicine, and general practice, each lasting 2 weeks. Additionally, students had the opportunity to choose two different weeks in any clinical specialty of their choice. The PY comprises 3 four-month rotations; surgery and internal medicine are compulsory and one specialty may be chosen freely. From January to the end of February 2023, a survey link was emailed to 423 medical students considered eligible, and three reminders were sent within the data collection period.

### Development of questionnaire and data collection

The questionnaire items were selected and adapted from existing surveys [5, 11, 15]. Novel items were implemented to monitor the specific circumstances of NSIs during the practical training placements within the CR and PY. The study collected data on the cumulative number of NSIs per student at the time of surveying, the reported reasons for the injuries, as well as students’ reporting practice. The survey also inquired into why NSIs were not reported. Furthermore, the survey investigated students’ knowledge regarding the risk of infection associated with NSIs and their understanding of the appropriate reporting procedures. Lastly, the participants were asked their opinions on the necessity of additional educational resources or offerings on the topics of preventing and managing NSIs.

The questionnaire utilized a combination of single-choice and multiple-choice options, as well as forced responses using binary and 5-step Likert scales ranging from 1 indicating “strongly disagree” to 5 indicating “strongly agree”. There was also the option to abstain. Free-text response questions were included to allow participants to add additional information.

### Data analysis

The survey data were exported and statistical analyses performed using IBM SPSS Statistics 28.0 and R 4.2.3 [21] with RStudio (Posit, Boston, Massachusetts, United States of America). Mean (M) and standard deviation (SD) were calculated for descriptive analyses. Odds ratios (OR) were calculated via logistic regression with the R package mfx [22] within R and transformed by the formula Q = (OR-1)/(OR + 1) [23] to return values between − 1 and 1, where 0 denotes no association/independency. A “thematic analysis” method was employed to summarize the open-ended responses from participants [24].

## Results

Out of the 423 medical students who were invited to participate in the study, a total of 84 students took part (19.6%). The average age of the participants was 26.7 years. Among the respondents, 65.5% were female (see Table 1). A large proportion of students were in their first and third rotation of the PY, a smaller number of respondents were enrolled in CR. Of note, 16.7% of students reported having completed training in the healthcare sector prior to starting their studies in medicine.

**Table 1:** Characteristics of participants (N/A = not available, as one response was skipped)

	*n*	%
**Medical students in clinical rotations and practical year**	84	100
**Gender**		
Female	55	65.5
Male	29	34.5
Diverse	0	0.0
**Completion of any training course in the healthcare sector**	14	16.7
**Type of training during which the injury occurred**		
CR	12	14.3
First rotation of PY	42	50.0
Second rotation of PY	1	1.2
Third rotation of PY	28	33.3
N/A	1	1.2

32.1% of respondents reported experiencing NSI during their clinical placements, numbering a total of 32 NSIs (see Table 2). The vast majority comprised puncture, cut, or scratch injuries (93.8%), while only a few suffered from contact with skin or some mucous membrane. On average, students experienced 1.38 NSIs. Among the respondents, more than a quarter reported at least one NSI.

**Table 2:** Frequencies of reported needle-stick injuries, placements, and specific circumstances

	*n*	%
**Frequency of NSIs**		
0 NSI	57	67.9
1 NSI	23	27.4
2 NSIs	3	3.6
≥ 3 NSIs	1	1.2
**Category of NSI**		
Puncture, cut, or scratch injury	30	93.8
Contact with skin or some mucous membrane	2	6.3
**Type of training during which the injury occurred**		
CR	10	31.3
First rotation of PY	15	46.9
Second rotation of PY	4	12.5
Third rotation of PY	3	9.4
**Wearing gloves at the time of injury**		
Yes	29	90.6
No	1	3.1
Missing	2	6.3
**Specialty of training during which the injury occurred**		
Surgery	15	46.9
Internal Medicine	7	21.9
Gynaecology and Obstetrics	5	15.6
Pediatrics and Adolescent Medicine	2	6.3
Other	2	6.3
General Practice	1	3.1

Most NSIs occurred during the first rotation of the PY, followed by CR. NSIs were most common in surgery, gynaecology and obstetrics, and internal medicine, as these placements in specialties were mandatory and most frequent. Expressed in terms of location, students primarily reported NSI at the university hospital (51.5%) and at academic teaching hospitals affiliated with their home university (12.5%). In 90.6% of cases, the students were wearing gloves at the time of injury.

The three most frequent activities associated with NSIs were identified as assisting in theatre, blood sampling, followed by suturing (see Table 3). More than half of the respondents described performing more than 10 invasive procedures/tasks per day at the time of injury. The primary reasons for injuries were given as lack of concentration, distraction, and other, undefined reasons. The other reasons for NSIs were identified from the seven open-ended responses provided. These included issues such as the distraction of operating personnel and their disregard for the safety of students. Injuries also occurred from transporting needles without adequate disposal facilities, often under incorrect assumptions regarding recapping mechanisms, or the lack of instrument trays and sharps bins to dispose of used needles or sharps. Additionally, coordination and communication errors during medical procedures were noted.

**Table 3:** Frequencies of needle-stick injuries categorized according to activity and reason

	*n*	%
**Activity**		
Assistance in theatre	10	31.3
Blood sampling	6	18.8
Suturing	5	16.5
Incorrect use of a safety device	3	9.4
On disposal of sharps	2	6.3
Insertion of a peripheral line	2	6.3
Puncturing or removal of a port catheter	2	6.3
Handing sharps to colleagues	1	3.1
Other	1	3.1
**Reason**		
Lack of concentration	21	65.6
Distraction	13	40.6
Other/miscellaneous	8	25.0
Stress/under time pressure	4	12.5
Lack of practice/instruction	4	12.5
Unavoidable situation (e.g. injury caused by third party or sudden patient movement)	3	9.4
Unknown instrument/unknown safety mechanism	3	9.4
Material defects	2	6.3
Work overload	2	6.3
Boredom through lack of a challenge	0	0.0
Lack of sleep	0	0.0

The majority of students reported the NSI to the correct contact person (see Table 4). In one-fifth of cases, the serological status of the involved patient was not determined, even though it was unknown. The most important reasons for not reporting a NSI were a fear of the consequences and viewing the injury only as minor; one-third stated that their supervisors viewed any report as unnecessary or that the patient’s serology was already known. Some students explained that the time required to report an incident was too great, or that they felt ashamed or uneasy, which prevented them from reporting.

**Table 4:** Reporting of needle-stick injuries in clinical rotation and practical year

	*n*	%
**Reporting**		
Yes	26	81.2
No	6	18.8
**Reasons for not reporting**		
Fear of consequences	3	50.0
Viewing the injury only as minor	3	50.0
Supervisor views any report as unnecessary	2	33.3
Patient’s serology was already known	2	33.3
Time required to report considered too great	1	16.7
Feeling ashamed or uneasy	1	16.7
Patient did not appear to be infectious	0	0.0
Lack of knowledge concerning reporting procedures	0	0.0
A reporting system was not in place or not available	0	0.0
The incidence report was forgotten	0	0.0
Doubts regarding the effectiveness of post-exposure prophylaxis	0	0.0
Fear the index patient tests positive	0	0.0
Fear of testing positive personally	0	0.0
Other	0	0.0

When queried on the appropriate course of action following NSIs, the majority of medical students correctly selected the necessity to contact the occupational health officer (90.5%). Regarding the infection risk associated with NSIs, students generally stated that they were well-informed (*M* = 3.9, *SD* = 1.2). Fewer students felt sufficiently trained in the handling of instruments for invasive procedures (*M* = 3.6, *SD* = 1.2). In terms of knowledge, 71.8% of the respondents were able to arrange the order of infection risk for HIV, HCV, and HBV correctly. Interestingly, 24.4% of the students rated the transmission risk of HBV higher than that of HCV.

Students were requested to state which training measures they would suggest to help with prevention of NSIs. More than one-fifth of the respondents (28.9%) expressed a desire for additional events focusing on the risk of NSIs and the proper handling of instruments. Among the suggested options (multiple responses were allowed), on-site instruction by the supervisor was generally selected (87.5%) as well as courses in the skills lab to train practical skills (50%), and training videos on the reporting procedure for NSIs on the learning platform of the university (33.3%).

Furthermore, the survey responses on reducing NSIs provided by students were summarized into four themes (Table 5). Altogether, these 12 responses added multifaceted views on strategies for NSI reduction: ‘Mindfulness and Training’ was the most mentioned (42%), stressing the need for focus and practice in high-risk situations and environments such as the operating room. ‘Communication and Reporting’ and ‘Infrastructure and Equipment’ each represent 25% of responses, highlighting clear communication within teams when performing high-risk procedures but also in situations in which NSIs have occurred as well as the importance of proper equipment and infrastructure. ‘Responsibility’ accounts for 8%, emphasizing team awareness.

**Table 5:** Summary of survey responses on proposed strategies for reducing NSIs

Theme	*n*	%	Exemplary quote
Mindfulness and training	5	42	“Improving focus during surgery and practicing often, especially under pressure, is key to lowering NSIs.”
Communication and reporting	3	25	“Open talk, truthful reporting, and right advice on NSIs are important for handling such situations well.”
Infrastructure and equipment	3	25	“Having enough disposal bins and safety instruments is very important.”
Responsibility	1	8	“Building a shared sense of responsibility and awareness in the team matters a lot.”
Total	**12**	**100%**	

**Fig. 1:**
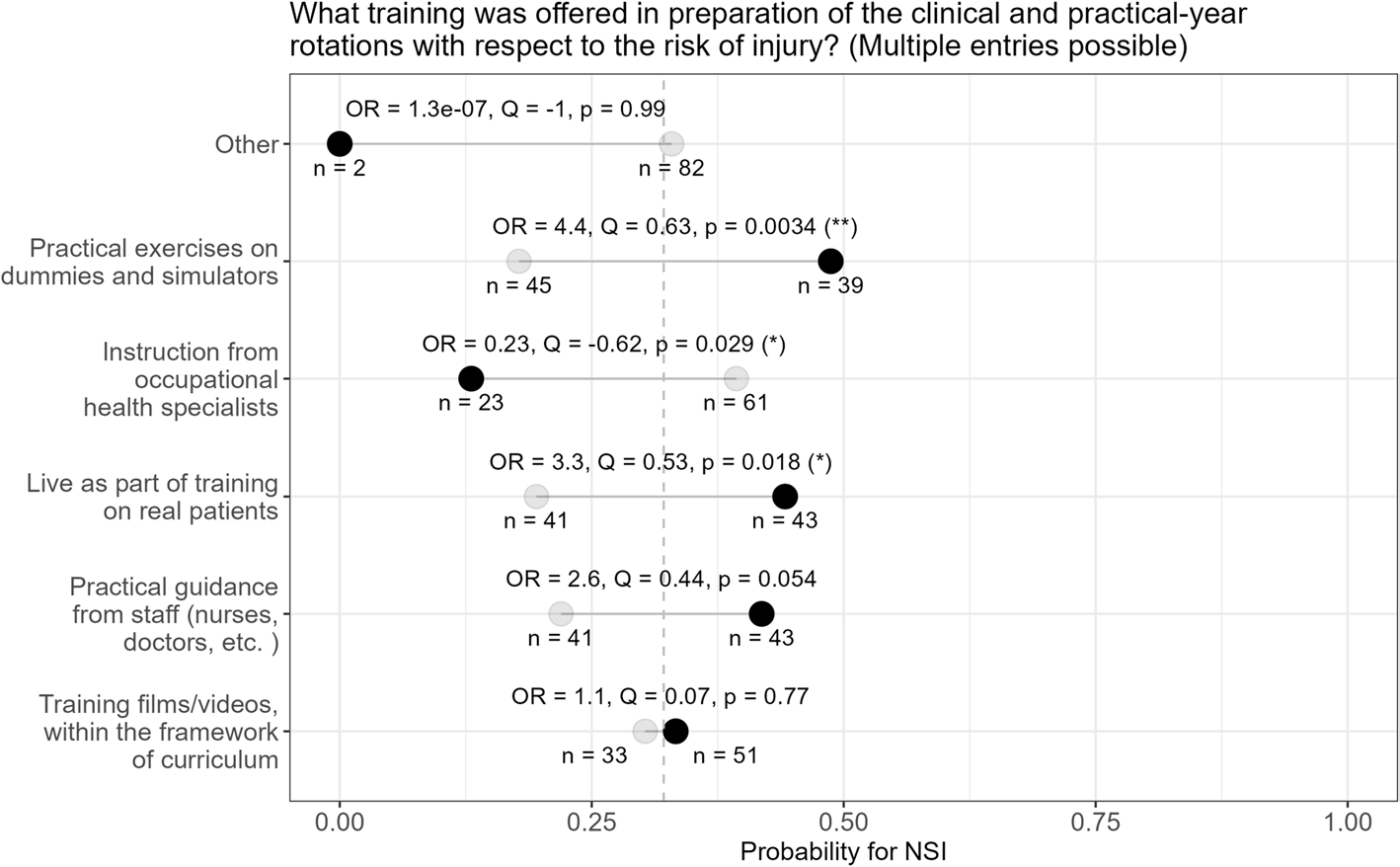
Training offered to students in preparation of practical activities and the associated probabilities of experiencing NSIs. Mean values for groups selecting (black circle) vs. not selecting (grey circle) the respective response, as well as overall mean value for the whole sample (dashed black line). Normalized odds ratios (Yule’s Q) quantify the strength of association

The association between the probability of an NSI occurring and the training offered in preparation of the clinical and practical-year rotations was found to be significant (see Fig. 1). Students who reported practicing on phantoms or simulators were found to be significantly more likely to experience injuries compared to those who did not receive such practical training. The same was evident when students received practical training on patients. Interestingly, students who received instruction from the occupational health officer beforehand, were less likely to experience NSIs.

## Discussion

### Frequency of NSIs among medical students

NSIs represent a serious health hazard for medical students, especially during workplace-based training. In our cross-sectional survey, 32.2% of the respondents reported suffering at least one NSI during their clinical placements (CR and PY). This is in line with the literature; NSI rates among medical students per year were reported to range from 14.8 to 29.5% [5, 11, 14]. In our survey, a significant number of reported injuries were observed during the CR and the first term of the PY, suggesting a potential correlation with the lower level of experience. This finding indicates a potentially high-risk phase at the beginning of workplace-based training, when invasive procedures are required but the necessary practical skills and routine may still be lacking. Interestingly, this contradicts another study, in which students reported 20.6% of the total NSIs at the beginning of their clinical rotations, which increased to 50.9% towards the end of the rotations [1]. Other studies determined a positive correlation between the frequency of performed invasive procedures and the level of ease in carrying out those procedures [25, 26]. This increase in familiarity might subsequently result in procedures being performed more frequently and a growing willingness to undertake complex procedures in challenging situations, which again might poses a higher risk of NSIs occurring.

Drawing comparisons between NSIs in medical students and physicians has proven challenging owing to the inconsistent data. Schmid et al. revealed that physicians suffered fewer than half the number of injuries in 1 year (11.1%) compared to medical students [14]. However, Wicker et al. reported that 55% of physicians had experienced at least one NSI during the last 12 months [3] which is higher than the average injury rate among medical students [5, 11, 14].

### NSIs related to specialty and student activities

In our study, NSIs occurred most frequently in the specialties of surgery, gynaecology and obstetrics, and internal medicine. This was confirmed in both national [1] and international studies [27–30]. In order to reduce NSIs in medical students effectively, it is crucial to conduct further studies to investigate whether the increased risk in specific specialties is solely attributed to a higher number of mandatory rotations involving invasive procedures or if other factors, such as lower risk awareness, high-stress environments, or inadequate supervision, also contribute to this risk.

Two activities in which NSIs occurred most frequently were related to surgery: assisting in theatre and suturing. Blood sampling, which is frequently performed by senior medical students in Germany, emerged as the third high-risk activity. In a study by Wicker et al., blood sampling and suturing were also the main culprits regarding NSIs, while in a study by Siegmann et al., assisting in theatre accounted for only 10.3% of all NSIs and suturing only 3.9% [1, 4]. Deisenhammer et al. found that procedures related to blood sampling were among the riskiest for medical students [31]. Internationally, phlebotomy plays only a secondary role owing to differences in the allocation of medical tasks. However, suturing and assisting in theatre are repeatedly described as the main activities responsible for NSIs in medical students [27, 32–34]. Unlike other studies [5, 15], disposal of sharps played only a minor role in our study. Of note, students from Würzburg underwent comprehensive training in basic skills in the beginning of their third year in medical school. The training sessions are conducted in the skills lab in Würzburg and focus on general safety measures including detailed demonstrations of the safety mechanisms incorporated into equipment and procedures. Of note, our study did not find any significant issues with basic protective measures, such as wearing gloves, suggesting that compliance with these measures was generally satisfactory. The majority of respondents (> 90%) were wearing gloves at the time of injury. This value is clearly above average compared to other studies reporting a rate of 43–70.4% [4, 35].

### Reasons for NSIs

With respect to the reasons NSIs occur, lack of concentration, distraction, stress/time pressure, and lack of practice/supervision played a prominent role in our study. Moreover, several students outlined a lack of communication and coordination among medical teams, especially in instances of needlestick injuries occurring in the operating room. This highlights the need for enhanced training for both medical students and staff in the workplace, especially in procedures in high-risk situations. Some students criticized unsuitable equipment and infrastructure that made the safe disposal of instruments difficult and increased the risk of needlestick injuries. Sharma et al. reported reasons such as time pressure (57%) and lack of skills (17%), followed by fatigue, unavoidable NSIs, and lack of assistance [34]. Students were also reported to have identified material defects as the third most common reason, in addition to time pressure and lack of experience [15]. Papadopoli et al. also described carelessness as the leading cause of NSIs, followed by lack of experience and personal reasons such as anxiety and fatigue [11]. Certainly, the reasons NSIs occur are diverse and multifactorial.

### Reporting procedure

Reporting plays a vital role in safeguarding health following a NSI. The NSI reporting rate among medical students in this study was above average at 81.3%, compared to other studies. Wicker et al. found in their study that only 28.7% of students reported all NSIs occurring, 20.9% reported their NSIs, and 50.4% did not report any NSI at all [4]. Other studies depicted reporting rates of 34–63% and non-reporting rates of 45% [5, 14, 15, 31]. In Würzburg, students were taught the reporting procedure through curricular lectures as well as mandatory advisory and screening sessions in occupational health services before the onset of practical training, which may explain the high reporting rate.

At the same time, further approaches must be developed in order to minimize the lack of reporting. The leading reasons for this were a fear of consequences and the viewing of the NSI as a minor injury, which is well in line with other studies [4, 34, 36]. In contrast, Salzer et al. described a lack of risk awareness among medical students as the main reasons, followed by guilty conscience and the belief that no high-risk patient was involved in the injury. Time pressure or a fear of negative consequences played only a subordinate role [15]. In summary, a lack of awareness of the risk as well as a fear of the consequences may contribute to medical students underestimating the significance of even minor injuries. Medical education currently appears to address these aspects inadequately. It is therefore crucial to prioritise the tasks of informing on and destigmatising NSIs, while also educating supervisors accordingly in their influential role.

### Students’ knowledge

This study found that 71.8% of medical students ranked the infection risks of HIV, HBV, and HCV accurately, indicating the extent of their knowledge on these transmissible diseases. However, there appeared to be some confusion among respondents regarding the correct HCV and HBV transmission rates. When Deisenhammer et al. asked medical students to estimate the transmission rates of HIV, HBV, and HCV, only 9% of first-year students and 45% of fifth-year students correctly assessed the risk of infection for HIV. The results were similar for estimations of HBV and HCV [31]. We therefore recommend refreshing knowledge on key pathogens such as HIV, HBV, and HCV prior to clinical rotations.

### Curriculum interventions

One third of our students suggested additional training measures toward the prevention of NSIs. In their free-text responses, they highlighted the importance of training, especially in high-stress scenarios, along with improved communication as key factors in reducing the occurrence of NSIs. The availability of enhanced practice opportunities is known to be associated with a reduction in injuries [35]. However, we discovered that participants in our study who underwent practical training on simulators and objects were more likely to suffer NSIs. We believe this is attributed to two factors. Trained students may be more experienced in handling invasive procedures on patients, which leads to a greater adoption of corresponding activities and thus an increased risk of injuries in absolute numbers. Additionally, a sense of security resulting from practical training led students to be less vigilant. Studies have revealed that simulation in training led to an increase in confidence for students [37–39]. Furthermore, it has be acknowledged that educational events do not necessarly lead to a realistic self assessment. Berger et al. compared the cardiopulmonary resuscitation (CPR) performance in two groups of medical students after two different curricular interventions. The problem-based learning group significantly overestimated their competence while performing CPR less effectively than the group simply trained [40]. Consequently, a highly formal and rigid approach to learning might not only hamper learning outcomes, but also foster an overestimation of abilities. Remarkably, our study found that instruction by the occupational health officer was associated with a lower probability of NSIs occurring. While practical teaching units focus on the precise execution of invasive procedures, instructions provided by occupational health specialists prioritize the context and associated risks. This approach may improve student awareness of the consequences and motivates active avoidance of NSIs.

In our cross-sectional survey, it remains uncertain at which point practical safety skills fall victim to carelessness, or when errors arise from inadequately trained procedures. Nonetheless, it is evident that both practical training and reflective practices are crucial for mindfulness and self-protection. Both must be incorporated into the strategy for curriculum development.

## Limitations

It is important to acknowledge certain limitations of our study. The study focused on a single medical school, thus limiting the range of experiences, and the conclusions may not be applicable to other medical schools or healthcare settings. NSIs can vary in nature and frequency, depending on geographical location, healthcare institutions, and patient populations.

Conducting this cross secional study with a specific cohort of medical students participating in clinical placements may limit the diversity and representativeness of the sample. Additionally, the response rate to the online survey was only 19.6%, potentially limiting the generalizability of our findings. While our study was designed as a quantitative survey, we never expected a high response rate given the nature of the subject. Additionally, the students surveyed were in their final years of study, a period typically marked by intense and demanding schedules. However, the reported numbers on the frequency of NSIs are consistent with previous studies. Moreover, we appreciate every participation, as the feedback reveals systemic issues and areas of improvement in regard to NSIs. NSIs have multifactorial causes, and each incident contributes to a better understanding and prevention of such occurrences. Regardless of representativeness, every incident should be taken seriously and ideally should not occur. Therefore, the qualitative insights of the study were of foremost importance. In this context, we decided against comparing subgroups (e.g., different levels of training), as the focus was on the overarching issue of NSIs rather than the specific characteristics or experiences of different groups.

It should be noted that participation in one rotation of the practical year was lower and thus may affect the statistical power and reliability of the study. However, we do observe international mobility of students towards the middle of the PY and this reduction in the participant numbers may be due to factors such as overseas stays impacting accessibility, along with varying levels of interest in and access to email communication .

## Conclusions

The findings of this study reveal that NSIs still pose a serious threat to medical students’ health. In order to reduce NSIs and encourage reporting, we believe the underlying reasons that were found in this study have to be addressed in the medical curriculum. In the future, teaching concepts to prevent NSIs, involving the supervisors in clinic, need to be established; their effectiveness should be investigated through subsequent surveys after implementation.

Based on our findings, we are going to develop a concise instructional video for medical students that provides information on the risks associated with NSIs and instructions on how to report such incidents. Additionally, to address issues relating to lack of concentration and a fear of consequences when reporting, we redesigned the practical module in the skills lab. In this module, senior medical students are trained to emphasise the hazards of inattentiveness during invasive procedures and to promote a focused approach. The module also includes discussions with participants on the reporting algorithm and potential consequences. Additionally, we shared the study results with clinic supervisors and the department overseeing health and safety at work and occupational health specialists, aiming to develop collaborative strategies and raise awareness of this issue. Further studies are required to evaluate the impact of these interventions on reducing NSIs among medical students and improving reporting behaviour.

## Data Availability

The datasets used and/or analysed during the current study are available from the corresponding author on reasonable request.

## Abbreviations

NSIs: Needle-stick injuries
CR: Clinical rotation
PY: Practical year
